# Perceptions of the importance of sports nutrition knowledge and barriers in implementing them: a qualitative study among track and field stakeholders in Sri Lanka

**DOI:** 10.1186/s40795-023-00817-7

**Published:** 2024-01-23

**Authors:** Ranil Jayawardena, Kalani Weerasinghe, Terrence Madhujith, Andrew P. Hills, Nishan Kalupahana

**Affiliations:** 1 Department of Physiology, Faculty of Medicine, University of Colombo, Colombo, Sri Lanka; 2 Department of Physiology, Faculty of Medicine, University of Peradeniya, Peradeniya, Sri Lanka; 3 Health and Wellness Unit, Faculty of Medicine, University of Colombo, Colombo, Sri Lanka; 4 Department of Food Science and Technology, Faculty of Agriculture, University of Peradeniya, Peradeniya, Sri Lanka; 5 School of Health Sciences, College of Health and Medicine, University of Tasmania, Newnham Drive, Launceston, TAS Australia

**Keywords:** Athletic, Knowledge, Perception, Sports nutrition, Sri Lanka, Stakeholder, Track and field

## Abstract

**Background:**

Integrating the core aspects of sports nutrition knowledge [SNK] into the multidisciplinary team is critical to improving an athlete’s performance and well-being. Conducting in-depth interviews with members of the sports-related team is a comprehensive method of gathering information on various aspects of SNK. This qualitative study aimed to examine the opinions and beliefs of stakeholders in athletics regarding the importance of SNK.

**Methods:**

Fifteen professional track and field athletes and stakeholders were recruited into the study. Separate in-depth interviews were conducted to collect information within four key themes. Practical difficulties in getting an appropriate meal were included in an additional theme. Thematic analysis was performed using NVIVO v10.0.

**Results:**

All participants were mindful of the importance of proper food habits for overall athletic outcomes and reported different opinions on meal timing and composition. The opinions on supplements were heterogeneous and both positive and negative claims were reported. Beliefs regarding hydration demonstrated that the cohort was well aware of the significance of adequate hydration plus the signs and consequences of dehydration with many reporting experiences of the negative consequences of dehydration. All respondents reported that both alcohol and smoking may have an adverse impact on performance and health.

**Conclusions:**

All respondents emphasized the importance of proper food habits for sports performance and well-being, but also identified barriers to optimizing nutrition.

**Supplementary Information:**

The online version contains supplementary material available at 10.1186/s40795-023-00817-7.

## Background

Proper dietary practices are considered an important determinant of athletic performance [1]. A personalized diet aims to enhance exercise capacity and performance, promote physiological adaptations to training, assist in recovery, manage weight, and ultimately improve well-being [2]. Saleh et al. conducted a seven-week nutrition education intervention via education programs and counselling on balanced dietary intake [3]and reported changes to body composition while increasing the efficiency of strength training. The combination of protein and carbohydrate led to a considerable reduction in muscle injuries and performance improvement. Therefore, athletes must learn what, when, and how to consume food and beverages before, during, and after training to optimize performance [2].

In addition to proper diet and hydration, according to a recent consensus report on SN [4], several ergogenic supplements have an impact on sports performance. Dietary supplementation with beta-alanine increases carnosine synthesis in skeletal muscle. Increased carnosine levels might increase exercise performance by several possible mechanisms [5]. Caffeine has been found in numerous studies to improve endurance by 2–4% at levels of 3–6 mg/kg/BW [6] and improve performance in a variety of endurance sports, including cycling [7] and running [8]. Creatine supplementation may speed up recovery between bouts of intermittent and continuous endurance-type exercise by reducing muscle damage and lost force-production potential [9]. A systematic review on acute beetroot juice supplementation showed improved performance in various time trials or increased time-to-exhaustion at submaximal intensities [10].

Dehydration may negatively influence muscle strength, power, and high-intensity anaerobic capacity [11]. Judelson et al. found that hypohydration consistently reduced strength [~ 2%], power [~ 3%], and high-intensity endurance [~ 10%] [12]. According to a meta-analysis, hypohydration decreases anaerobic power by -5.8 ± 2.3% and muscle strength by 5.5 ± 1.0% [13].

The articulation of knowledge among disciplines is crucial for better sporting performance and achievements, as this integration increases the understanding of the emotions and behaviours of athletes involved in competitive sports at the elite level [14]. A team comprised of expert professionals from various fields, such as physical therapists, psychologists, doctors, nutritionists, and physical education professionals, not only enables athletes to improve technically but also allows for the investigation of psychological phenomena and nutritional status that might improve the athletes’ performance [15].

Researchers acknowledge the significance of qualitative research as the best approach to investigating phenomena that are challenging to measure, where processes must be evaluated, knowledge about a culture is limited, or where reasons for outcomes must be determined [16]. A recent qualitative study with twelve endurance athletes to explore athletes’ experiences of RED-S [relative energy deficiency in sports] found that system-wide educational prevention and awareness interventions are critical for athletes and support personnel such as coaches, parents, dieticians, psychologists, and sports medicine staff [17].

According to the findings, system-wide educational prevention and awareness interventions for athletes and support personnel such as coaches, nutritionists/dieticians, psychologists, and sports medicine staff are crucial. Similarly, another qualitative exploration was conducted to investigate the context in which personal trainers provide nutrition care, with the goal of better understanding personal trainers’ perceptions of nutrition care and their role and scope of practice [18]. Semi-structured telephone interviews with 15 personal trainers concluded that, despite concerns about competence, their nutrition care practices had not changed [18]. Jessyca and the team conducted another quantitative analysis to characterize the recovery experiences of female collegiate athletes who have suffered from eating disorders [19] and employed semi-structured interviews to record recovery stories and factors that initiated, assisted and hindered recovery [19].

It can be concluded that when dealing with elite-level athletes, integrating the core aspects of sports nutrition knowledge [SNK] of all team members in the multidisciplinary care team is critical to improving the athlete’s overall outcomes. Conducting in-depth interviews with members of the sports-related multidisciplinary team can also be a comprehensive method of gathering information on various aspects of SNK. Therefore, this qualitative study aimed to examine the opinions and beliefs of various stakeholders, including athletes, support staff such as team doctors, sports nutritionists, physiotherapists, masseurs, and university academics, regarding the importance of SNK for athletes.

## Methods

### Design and study population

Whilek and field athletes registered under the Sri Lankan Athletic Association, participating in events such as sprinting, long-distance running, throwing, and jumping, were invited to the study. Athletes with a current injury lasting more than six months or a history of doping abuse were excluded. The selection process involved approaching a diverse range of professionals, including sports medicine doctors, sports nutritionists, dieticians, university academics specializing in sports sciences, sports psychologists, national-level sports coaches, and senior administrators from the Ministry of Sports, Sri Lanka. Support staff were recruited based on a minimum of two years of experience working closely with track and field athletes. Invitations were extended to individuals from institutions like the Institute of Sports Medicine, Sri Lanka, the Ministry of Health, Sri Lanka, and local universities. While recruitment was selective, the saturation of ideas achieved during interviews ensured a comprehensive understanding of the study’s objectives. Interviews were conducted with the first responders among the invited track and field athletes and support staff, selected based on their availability and willingness to participate. The detailed guide used for in-depth interviews is attached (Supplementary Material 1).

Informed written consent was obtained from each participant after an in-depth explanation of the study and giving them adequate time to ask questions and clarify doubts about the research. Further, verbal consent was recorded during the interview. To ensure the anonymity of patients or participants, we have refrained from including specific age and sex information in the tabulated characteristics of each athletic stakeholder. Instead, age ranges have been provided in conjunction with other non-identifiers (Tables 1 and 2). All methods were performed in accordance with the Declaration of Helsinki, and ethical approval for the study was obtained from the Ethics Review Committee, Faculty of Medicine, University of Peradeniya, Sri Lanka [Ref No. 2022/EC/66].

**Table 1:** Details of the professional athletes

Participant ID	Age range	Sport	Level of education	Highest achievement
A_1_	20–29 y	High jumper	A/L	SE
A_2_	20–29 y	Javelin thrower	A/L	SE
A_3_	20–29 y	Marathoner	O/L	SE
A_4_	20–29 y	Sprinter	A/L	An Olympian

SE = Super elite (with national records of 1, 2 and 3), O/L = Ordinary level up to grade 11, A/L = Advanced level up to grade 13

**Table 2:** Details of the expert support staff

Participant ID	Age range	Occupation	Level of education	Experience in sports (y)
O_1_	30–39 y	Masseur	A/L	9 y
O_2_	30–39 y	Sports nutritionist	MD	3 y
O_3_	30–39 y	Physiotherapist	BSc	8 y
O_4_	30–39 y	PT teacher	Dip in teaching	7 y
O_5_	30–39 y	Sports dietician	MSc Nutrition	2 y
O_6_	30–39 y	Sports psychologist	MSc Sports Exercise Psychology	3 y
O_7_	30–39 y	University academic	MSc Sports Science	5 y
O_8_	40–49 y	Administrator	A/L	30 y
O_9_	40–49 y	Coach 1	A/L, IAAF level 2 coach	15 y
O_10_	40–49 y	Coach 2	A/L	25 y
O_11_	40–49 y	Nutrition doctor*	MD	11 y

*Nutrition doctor (refers to the sports medicine physician in the country of interest)

PT = Physical training, SN = Sports nutrition, A/L = Advanced level up to grade 13, MSc = Master of science., MD = Doctor of Medicine, BSc = Bachelor of Science

### Data collection

Data were collected via face-to-face computer-mediated in-depth interviews [platform: Zoom Video Communications, Inc., California, USA]. Each participant was separately interviewed following a pre-designed protocol. The principal investigator [RJ] conducted in-depth interviews using a set of open-ended, semi-structured questions to guide the participants and maintain consistency across all participants. The interview comprised four themes of questions regarding participants’ opinions on (1) food habits associated with training, (2) intake of sports supplements, (3) hydration, and (4) other habits such as the consumption of alcohol and smoking. If additional information was raised, it was included in another additional theme.

Each in-depth interview lasted about 20–60 min, and participants’ responses were recorded. The in-depth interviews were conducted either in Sinhalese or English. Two research assistants were also logged into each interview with the facilitator [RJ] as observers and noted important comments on the subject at each in-depth interview. RJ was responsible for providing guidance, maintaining focus, stimulating constructive discussion, and enduring time constraints while maintaining a neutral stance on the contents of the discussion. At the end of each session, RJ and the observers summarised the key points.

### Data analysis and rigor

NVIVO v10.0 (QSR International, Southport, UK) was employed to conduct a thematic analysis on the qualitative data collected. This analytical process involved a systematic approach, beginning with the preselection of themes based on the study’s objectives. Subsequently, responses from each participant were meticulously coded and organized under their respective themes within the NVIVO software. This coding process was iterative, with careful consideration given to the nuances and depth of participant responses. Theme development was carried out through an in-depth examination of coded segments, facilitating the identification of patterns and relationships within the dataset.

The themes selected for SNK encompassed their ideas and beliefs regarding (1) athletes’ food habits, (2) sports supplements, (3) hydration, and (4) other habits (alcohol and smoking). Extra information was not directly aggregated under the above themes and was clustered as a new theme.

RJ and two observers collectively analyzed participants’ verbal responses in each in-depth interview using notes and saved recordings. This process took place immediately following each interview, where they recorded all facts mentioned by participants and cross-referenced their notes with the tape recording. Each in-depth interview resulted in a comprehensive document, collectively assessed by the research team at the study’s conclusion. Initially recorded in Sinhalese, the native language of respondents (except for two participants), independent translators later translated the cumulative interviews into English. The research team, with translator assistance, compared each translation through an iterative consensus procedure to produce the final written piece.

## Results

### Socio-demographic characteristics

Fifteen participants were involved in the current study [*n* = 15], including four track and field athletes and eleven expert support staff. The non-identifying characteristics of the participants have been presented in Tables 1 and 2. The four elite-level athletes were involved in four different track and field categories, and all had the highest achievement at the super-elite [SE] level, i.e., with national-level medals or above. In addition, the support staff covered areas such as sports medicine, SN, sports psychology, physiotherapy, physical training, and sports administration. The thematic analysis of interview data yielded four key themes, and the key findings of each theme are presented in Table 3.

**Table 3:** Summary of major themes and key findings

Theme	Key findings
Theme 1: Importance of Food Habits	- Highlighting the significance of proper food habits for sports performance and overall well-being - Emphasizing pre-training meals, a variety of foods including sandwiches, buns, bananas, and milk tea were highlighted - Noting a lack of knowledge on portion sizes and timing before training - Underscoring the importance of meals during and after training, with post-training meals predominantly starchy - Recommending early post-training meals with both carbohydrates and proteins, as advised by coaches
Theme 2: Beliefs about Sports Supplements	- Reporting positive effects by some, while others expressed doubts about their efficacy - Pointing out instances of adverse effects and improper usage - Noting that many obtained supplements without proper medical advice - Commonly mentioned supplements included whey protein, creatine, and pre-workout products
Theme 3: Opinions about Hydration	- Recognizing the importance of hydration for performance and recovery - However, reported firsthand other experiences of dehydration during training and competition - Identifying various strategies to overcome dehydration - Using different methods such as urine colour and weight measurement to identify dehydration.
Theme 4: Perceptions about Other Habits	- The majority discouraged alcohol consumption, and smoking was universally rejected - Nevertheless, alcohol consumption among elite athletes was not uncommon - Emphasizing adequate sleep for recovery, despite reported poor sleeping habits
The additional Theme	- Attributing poor dietary habits to a lack of knowledge and practical difficulties - Mentioning factors such as a lack of suitable places to eat, economic crises, and food insecurity as the barriers to getting an appropriate meal

### Theme 1: importance of food habits

All participants [15/15] identified that proper food habits are important in determining sports performance, recovery, reducing injury risk, and well-being. Food habits in this context were categorized into pre-training, during training and post-training meals.

Resembling pre-training meals, an athlete stated that;

*“Yes, it is hard to run without food.”* [Marathoner, 20–29 years].

Sandwiches, buns, bananas, fruit juice, rice flakes, and milk tea were reported to have been consumed or advised to be consumed before training sessions.

*“I am taking a bun as I get energy sooner. Sometimes I take milk tea and a banana before training.”* [Marathoner, 20–29 years].

*“Generally, they take a banana before the training. Some take plain tea and a bun.”* [Masseur, 30–39 years].

However, a senior athlete said,

*“I eat rice before training; it is not a huge meal, generally rice with one vegetable.”* [Sprinter, 20–29 years].

Although they accurately mentioned carbohydrate-rich foods as pre-training meals, none had any idea of portion size or the time gap before training.

The majority of participants [13/15] stated the importance of taking a meal during the training as well.

*“During the training, I asked to eat bananas and dates.”* [Coach 1, 40–49 years].

However, meals during the training were not consistent.

*“Generally, I do not feel hungry on some days; if I get hungry, I will eat, otherwise I won’t.”* [Marathoner, 20–29 years].

They didn’t have a clear idea of the portion sizes or frequency of intake according to training duration and intensity, and most of them took the meal only out of hunger.

All athletes were taking a post-training meal, which is primarily a starchy meal.

“I’m taking some boiled chickpeas to eat, some types of yams such as sweet potatoes, and finger millet porridge to eat after training” [Sprinter, 20–29 years].

Their coaches instructed them to have a meal as early as possible.

“After the training, I advised them to take their meal within half an hour.” [Coach 1, 40–49 years].

Almost all post-exercise meals were predominantly starchy foods. However, sports nutritionists and sports medicine physicians mentioned the importance of having both carbohydrates and proteins after training.

*“After the training, some snacks should be taken as soon as possible, furthermore, it is better to have a meal with both carbohydrates and proteins, such as a chicken sandwich.”* [Sports nutritionist, 30–39 years].

*“It is recommended to eat within half an hour after training, which should contain both proteins and carbohydrates.”* [Sports medicine physician, 40–49 years].

### Theme 2: beliefs about sports supplements

The perceptions regarding sports supplements were diverse within the study group, revealing varied beliefs about their impact on performance. Some participants expressed positive experiences:

*“When I take supplements after strength training, I feel a better recovery.”* [High jumper, 20–29 years].

However, heterogeneous ideas were also present, with a physical training teacher expressing disbelief in the efficacy of supplements:

*“It is purely psychological; nonetheless, it does not affect their performance.”* [PT teacher, 30–39 years].

Furthermore, the study revealed common supplements mentioned by participants such as whey protein, creatine, and pre-workouts. While not exhaustive, this provides a qualitative insight into the supplement landscape discussed during the interviews.

Additionally, some athletes acknowledged inconsistent supplement use:

*“They don’t even consume protein before or after working out. They take when they remember.”* [Javelin thrower, 20–29 years].

Concerns about adverse effects were raised, such as a javelin thrower sharing an experience with a pre-workout supplement:

*“I’ve seen cases where taking supplements resulted in a decrease in performance. Movements are reduced due to body bulking due to high protein supplements. In addition, I took a pre-workout supplement, that had a high caffeine level, which adversely affected my performance.”* [Javelin thrower, 20–29 years].

Furthermore, it was observed that athletes often obtained supplements without proper medical advice, relying on shopkeepers’ recommendations or observing high-performing athletes. Many athletes expressed reluctance to share their experiences with supplements, reflecting a nuanced perspective within the study group.

### Theme 3: opinions about hydration

In contrast to sports supplements, participants universally stressed the paramount importance of hydration for sporting performance, recovery, and injury prevention, recognizing it as a fundamental aspect of sports nutrition. Commonly consumed beverages during training included water, oral rehydration solutions *(jeewani)*, king coconut water, and isotonic drinks.

The nutrition doctor underlined the significance of monitoring water intake during training. The term ‘nutrition doctor’ in this context refers to the sports medicine physician in the country of interest, who emphasized the significance of sports nutrition during training.

*“If they don’t get water during training, their ability to stretch will be lower.”* [Nutrition doctor, 30–39 years].

Despite being aware of the adverse consequences of dehydration, participants shared personal experiences, ranging from moderate to severe symptoms:

*“If I don’t drink enough water on my rest day, I can’t run on the next; We might experience cramps, muscle tightness, and thirst while working out.”* [Sprinter, 20–29 years].

Some participants observed runners losing balance due to inadequate hydration:

*“They experienced losing balance when running, including running in a zig-zag manner.”* [PT teacher, 30–39 years].

Extreme situations, such as vomiting and unconsciousness, were also described.

Participants highlighted various techniques used to identify dehydration within the athlete population, including assessing urine colour and measuring body weight:

*“To gauge hydration levels, we monitor urine colour, suggesting that a dark colour indicates limited body water.”* [PT teacher, 30–39 years].

*“I teach them to check pre- and post-weight and calculate water loss.”* [University academic, 30–39 years].

Additionally, the physiotherapist introduced a unique technique for identifying dehydration:

“*I use the skin test basically to identify dehydration*.” [Physiotherapist, 30–39 years].

### Theme 4: perceptions about other habits [alcohol and smoking

The majority [12/15] of participants expressed the view that refraining from alcohol is essential for athletes:

*“Both alcohol and smoking are not good habits, and alcohol is disadvantageous for recovery.”* [Physiotherapist, 30–39 years].

However, some support staff who work closely with athletes have had different experiences with alcohol consumption.

*“Most of them take alcohol, which is not a habit. But they take it when they attend a party.”* [Masseur, 30–39 years].

*“Some stay number one in Sri Lanka even though they have the practice of taking a shot [alcohol] in the evenings.”* [Administrator, 40–49 years].

However, none accepted smoking. Sport psychologists might think it is for coping.

*“Smoking can be a coping mechanism, which is not a great thing to do.”* [Sports psychologist, 30–39 years].

In addition to alcohol and smoking, they identify sleeping habits as fundamental for recovery and performance.

*“Both coaches and athletes should obtain adequate sleep.”* [Administrator, 40–49 years].

*“They should sleep at least 8 hours removing all the barriers to getting a good sleep.”* [Coach 2, 30–39 years].

Although sufficient sleep is recognized as an essential practice for recovery and general well-being, poor sleeping habits were identified among athletes for a range of reasons, such as spending time on social media and other commitments such as education and travel.

### Additional theme: practical difficulties in getting an appropriate meal

It is reported that both lack of knowledge and practical difficulties are the main reasons for poor dietary habits among athletes in Sri Lanka. An experienced sports dietician once mentioned that.

*“Mainly because of the lack of knowledge, not only of the players but also of the coaches. Furthermore, it is valuable to educate both players and coaches on SN concepts.”* [Sports Dietician, 30–39 years].

*“Lack of knowledge was the main issue that I observed.”* [Sports medicine physician, 40–49 years].

However, several mentioned the practical difficulties of finding nutritious foods. According to a senior lecturer in sports science, this was significant for college athletes as well.

*“What I have seen is that the university athletes don’t get a place to eat early in the morning.”* [University academic, 30–39 years].

When they have training twice a day, their evening meal has been affected due to these practical issues.

*“I’m first eating the food that is brought from home after the morning session, but I have to take lunch from a restaurant [where I can’t choose much] before evening training.”* [High jumper, 20–29 years].

Furthermore, they don’t have a suitable place to cook or buy food when they come to the ground. Both players and officials acknowledge that the current economic crisis in the country results in food insecurity as well.

## Discussion

### Novelty of the findings

To the best of our knowledge, this is the first qualitative study to evaluate the perception of the importance of SNK among athletic stakeholders in Sri Lanka. This study is unique because we interviewed both athletes and support staff to obtain a comprehensive picture of the perception of the importance of SNK among athletes.

### Salient findings

Our study revealed important insights into the nutritional knowledge of elite athletes. In Theme 1, we found a clear need for updated sports nutrition training programs for athletes and their support teams. This emphasizes the importance of improving educational efforts in this area. Additionally, stakeholders consistently stressed the importance of having appropriate food and beverages available during different stages of training.

Moving on to Theme 2, there was a strong consensus on the role of sports nutritionists in prescribing all dietary and ergogenic supplements. This highlights the crucial role these professionals play in optimizing athletes’ nutritional strategies.

Theme 3 drew attention to the pressing need for increased awareness of dehydration signs and symptoms. It was recommended to make effective rehydration solutions easily accessible for athletes. Theme 4 highlighted a general aversion to alcohol consumption among competitive athletes. There was unanimous agreement on the importance of promoting awareness of proper sleep hygiene. However, none accepted smoking, with the interviewed sport psychologist suggesting that it may represent a coping mechanism.

An additional theme emphasized the financial support necessary to sustain athletes’ dietary habits and cover essential training-associated expenses. These key findings collectively provide a nuanced understanding of nutritional knowledge, offering practical recommendations for the multidisciplinary care team dedicated to supporting elite athletes.

### Potential mechanisms for the findings

Athletes utilize a range of dietary approaches to maximize glycogen stores during training sessions and enhance performance. The post-exercise period is often regarded as the most significant section of dietary intake timing. Sports nutritionists should collaborate closely with coaches and athletes, ensuring that athletes consume the correct types, amounts, and timing of snacks following training and competitions. In general, Sri Lankans consume only 10% of their calories from protein and over 70% of their calories from carbohydrates [20]. Similar practices might exist in the athletic population also, moreover, protein-rich foods are expensive, and in Sri Lanka’s current financial crisis, many athletes are unable to afford a protein-rich post-workout meal, resulting in food insecurity [21]. Besides that, a variety of different stakeholders might encounter difficulties when attempting to translate complex information into contextual and usable performance strategies [22].

Many professional and amateur athletes use supplements to meet higher exercise-induced nutrient needs, match nutritional recommendations, and/or improve athletic performance [23]. Supplements were used by 91.5% of Sri Lankan athletes, with a considerable percentage of multivitamins [51.8%], creatine [37.3%], and protein [14.8%] users consuming them without any scientific basis [24]. Some supplements may assist athletes, but misusing them may harm their health, performance, livelihood, and reputation [if an anti-doping rule violation occurs] [25].

It is well-recognized that hydration influences performance, injury prevention, and recovery [26]. While several recommendations have been developed to assist with fluid intake and its timing [i.e., before, during, and after exercise], research has shown that approximately 70% of collegiate athletes arrive at practice dehydrated [27]. Education, accessibility, experience, and palatability can all change behaviours associated with hydration [28]. The majority of the athletic stakeholders tested in the current study mentioned water, *jeewani*, fruit juices, especially watermelon and king coconut water, and rarely sports drinks or isotonic solutions, as rehydration strategies. Interestingly, this can be considered a positive sign regarding their SNK on hydration because a recent randomized controlled trial showed that oral rehydration solutions were more effective than sports drinks and water at replacing the fluid deficit during exercise recovery [29]. Many players performed better in the endurance exercise time trial performance using an oral rehydration solution and sports drink than with water [29]. While hydration can be assessed early using appropriate methods, crude estimates of hydration status include vital signs and sensations of thirst and dryness in the mouth [30].

Empirical evidence illustrates that athletes are at heightened risk for hazardous alcohol consumption compared with their non-athletic peers [31]. A meta-analysis revealed that student-athletes had a lifetime prevalence of alcohol consumption of 78% [32]. Almost all the respondents in this study commented on how crucial it is for any athlete to abstain from alcohol [33]. Even though smoking is considered detrimental to anyone’s health, a growing body of literature has reported an increase in nicotine use among young athletes [34]. Smoking prevalence among Finnish Olympic athletes was 11.4%, while about 25% of college athletes in the United States were found to smoke [35]. The majority of smokers indicated potential adverse effects, whereas non-tobacco users also reported detrimental effects on performance [34].

With regard to physical development, emotional control, cognitive function, and quality of life; sleep is also a crucial component. Increased sleep length and better sleep quality in athletes may be linked to better performance and competitive success, in addition to being crucial for the recovery and adaptation processes between workout sessions [36]. The American Academy of Sleep Medicine states that adult athletes require between 7 and 9 h of sleep for optimal performance and health, while adolescents should get more sleep, ideally between 8 and 10 h [37]. Despite this, poor sleeping habits were identified among athletes in the current study due to many reasons, such as spending time on social media and other commitments including education and travel.

### Key recommendations

**Fig. 1:**
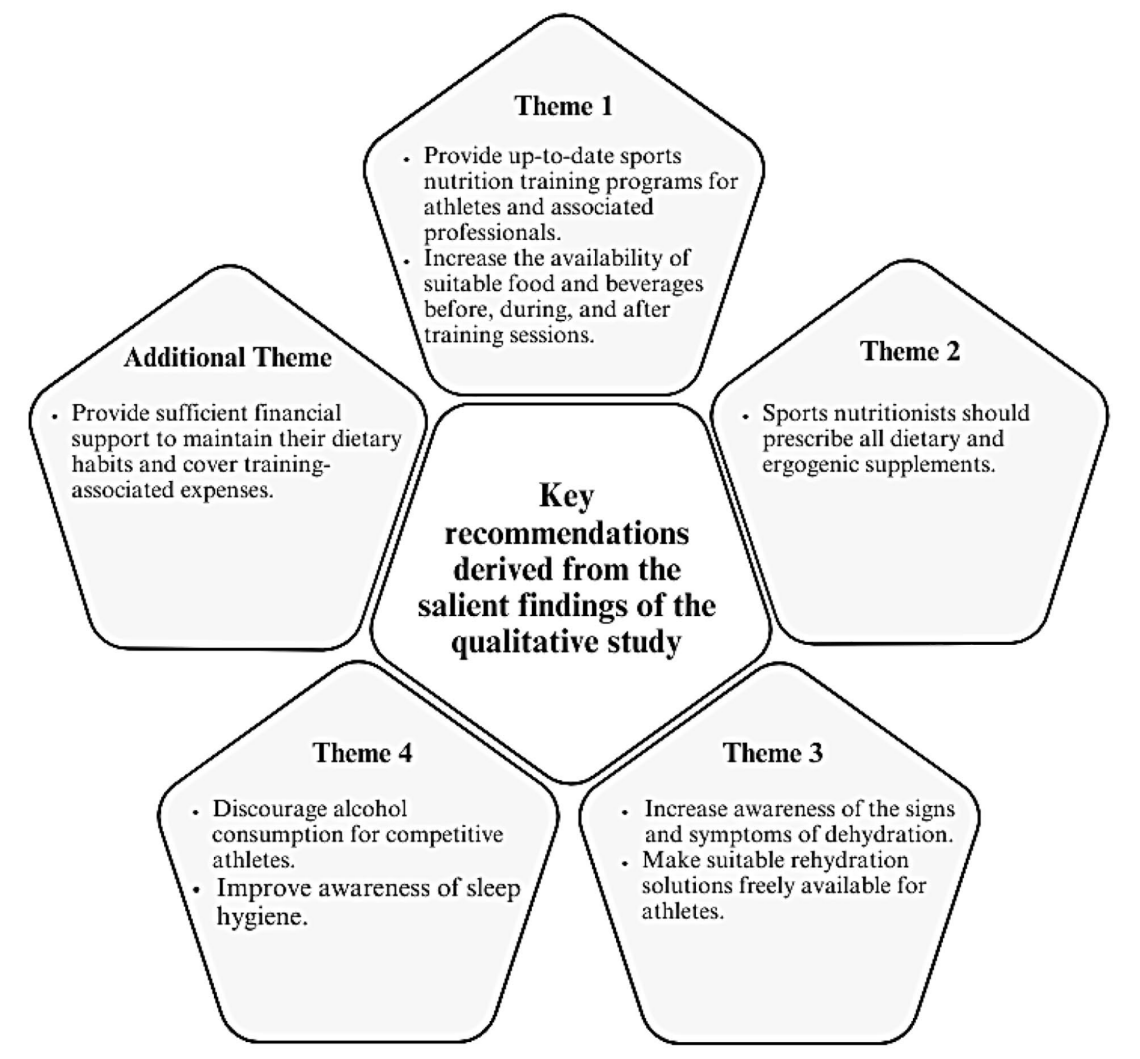
Key recommendations derived from the salient findings of the qualitative study

Figure 1 illustrates the key recommendations derived from these salient findings. The focus on these varied perspectives addresses the identified research gap and underscores the essential role of holistic sports nutrition practices in optimizing athlete outcomes.

### Limitations

One notable limitation of this study was the inclusion of only one athlete per event, due to the practical constraints such as busy training schedules, frequent overseas travel, and challenges in securing elite athletes. Despite extending invitations to a significant number of successful athletes, the study’s sample size remained constrained. Recognizing the importance of a larger and more diverse sample for the generalizability of findings, it is acknowledged that future work with a larger participant pool would be beneficial. In addition, the current study did not incorporate physical interviews which would have been more interactive. However, the fact that participants are scattered all over the country makes it challenging to conduct in-person interviews. Another potential limitation is that the scope of the current work was limited to four key nutrition themes; other important nutrition-related topics such as dietary preparation for different tournaments, carbohydrate loading, food label reading, and doping, should be considered in future research.

### Strengths

A key strength of the present study was that we included both support staff and athletes, and we received a very comprehensive overview of their opinions regarding SN topics. We invited key officials from each profession related to athletics in the country; accordingly, we may expect that the answers were representative. Qualitative research is crucial for identifying ground-level information and our findings may be useful for both practitioners and policymakers to make informed decisions to improve SN among athletes. Although dietary patterns vary between countries, our findings could also be beneficial to other Asian athletes, particularly Indian athletes. It is important to develop and validate culturally specific SNK tools to access baseline SNK in this population and identify the gaps. More broadly, research on culturally specific nutritional interventions is useful to improve SNK and its performance.

In conclusion, all respondents identified proper food habits, including pre-, during, and post-meals, as important in determining sports performance, recovery, and maintaining good health and well-being. However, the opinions of the support staff on supplements were heterogeneous, and both positive and negative claims on performance were reported. Our findings on hydration revealed that, while they indicated various rehydration strategies, signs of dehydration, and adverse effects of dehydration, many have experienced moderate to severe dehydration symptoms. Almost all the respondents stated that alcohol are unhealthy habits. None accepted smoking, with the interviewed sport psychologist suggesting that it may represent a coping mechanism. All the respondents stated the importance of maintaining good sleep hygiene for recovery and performance Importantly, the majority of respondents expressed that unhealthy dietary practices are incompatible with improving sports performance. Their perspectives highlighted the key contributing factors to unhealthy dietary behaviours, including a lack of nutritional knowledge, practical difficulties, and the prevailing economic crisis in the country.

### Electronic supplementary material

Below is the link to the electronic supplementary material.

Supplementary Material 1

## Data Availability

Not applicable.

## Abbreviations

LEA: Low Energy Availability
RED-S: Relative Energy Deficiency Syndrome in Sports
SE: Super-Elite
SN: Sports Nutrition
SNK: Sports Nutrition Knowledge
