# Supplementary material for: Perceptions of the importance of sports nutrition knowledge and barriers in implementing them: a qualitative study among track and field stakeholders in Sri Lanka
Source: BMC Nutr. 2024 Jan 23;10:17. doi: 10.1186/s40795-023-00817-7 (PMC10804860; doi:10.1186/s40795-023-00817-7)
Supplement: Supplementary file 1 — Supplementary Material 1 [file 40795_2023_817_MOESM1_ESM.docx]

**A guide to the in-depth interview**

**Introduction**

Thank you for providing us with your valuable time by participating in this interview. Today, we will be discussing your opinions and beliefs on different sports nutrition topics relevant to Sri Lankan athletics. Through this study, we expect to explore the view of the gap in sports nutrition knowledge in the Sri Lankan athletic population. We want to hear your opinions and remember that there are no right or wrong answers. Because this is a qualitative study, the probes may change based on their responses to the main questions. You are free to answer the questions in any way you feel comfortable. You can refuse to answer if you do not want to answer at all. If there is any unclear question, do not hesitate to clarify and make us explain more about it. We are planning to keep a record of this conversation to enable us to clarify unclear areas of this discussion later; we hope that you will consent to this. We guarantee that what is said will be kept strictly confidential.

Be comfortable- we hope you will find the session interesting and enjoyable.

**Respondent register**

Date:

Time:

Name of the moderator:

Participant details:

- 1. *Name:*
  2. *Occupation/ position:*
  3. *Age:*
  4. *Gender:*
  5. *Level of education:*
  6. *Experience in sports:*

**Qualitative assessment of the importance of sports nutrition for athletics**

**Theme 1- Opinions about food habits**

**Question: Why are proper food habits important for athletes?**

Probes

1. What are the main macronutrients in foods and provide some examples?
2. What are the advantages of taking a pre-training meal and provide some examples?
3. Do you suggest any foods or beverages during the training? If so, give some e.g.
4. What are the advantages of taking a post-training meal and provide some examples?
5. What are the practical difficulties of getting an appropriate meal?

**Theme 2- Opinions and beliefs about the sports supplements**

**Question: What is your opinion on sports supplements?**

Probes

1. What sports supplements have you taken or recommended?
2. Do you think taking sports supplements is effective for athletics?
3. Are there any beliefs or myths among athletes related to the intake of supplements?
4. Have you experienced any adverse effects from taking sports supplements?

**Theme 3- Opinions about hydration**

**Question: Why do you think hydration is important for athletics?**

Probes

1. How do you identify dehydration?
2. What are the negative consequences of dehydration?
3. What are the beverages that you must drink to hydrate yourself?

**Theme 4- Opinions about the other habits (alcohol and doping)**

**Question: Why do you think athletes should follow certain habits?**

Probes

1. What is your opinion on the consumption of alcohol?
2. What is your opinion on smoking?
3. Any other behaviours do you believe are particularly important for athletics?
4. Are there any other practices that athletes should follow during competitions?
